# 

**Published:** 1903-11

**Authors:** 


### BOOKS RECEIVED.

The Practice of Obstetrics. Designed for the Use of Students and Practitioners of Medicine. By J. Clifton Edgar, Professor of Obstetrics and Clinical Midwifery in the Cornell University Medical College; Attending Obstetrician to the New York Maternity Hospital. Royal octavo, pp. 1111. With 1221 illustrations, many of which are printed in colors. Philadelphia: P. Blakiston's Son & Co. 1903. [Price: cloth, $6.00; sheep or half morocco, $7.00.)

The Practical Medicine Series of Year Books. Ten volumes. Issued under the general editorial charge of Gustavus P. Head, M. D., Professor of Laryngology and Rhinology in the Chicago Post-Graduate Medical School. Volume X. Skin and Venereal Diseases, Nervous and Mental Diseases. Edited by W. L. Baum, M.D., Hugh T. Patrick, M.D. Duodecimo, pp. 236. Chicago: The Year Book Publishers. 1903. (Price, $1.25; entire series, $7.50.)

A Textbook of the Diseases of Women. By Thomas A. Ashby, M. D., Professor of Diseases of Women in the University of Maryland. Octavo, 661 pages, with 233 illustrations. Baltimore: Williams & Wilkins Co. 1903.

Consumption a Curable and Preventable Disease. What a layman should know about it. By Lawrence F. Flick, M. D., Founder of the Pennsylvania Society for the Prevention of Tuberculosis. Duodecimo, 295 pages. Philadelphia: David McKay, Publisher. 1903. (Price, $1.00.)

A Manual of the Practice of Medicine. By A. A. Stevens, A.M., M.D., Professor of Pathology in the Woman's Medical College of Pennsylvania. Sixth Edition, thoroughly revised, enlarged and reset. Handsome post-octavo of 556 pages, illustrated. Philadelphia, New York, London: W. B. Saunders & Company. 1903. (Flexible leather, $2.25 net.)

The Principles and Practice of Hydrotherapy. A Guide to the Application of Water in Disease. By Simon Baruch, M.D., Professor of Hydrotherapeutics in the New York Post-Graduate Medical School and Hospital. Octavo, pp. 506; illustrated. New York: William Wood & Co. 1903. (Price, $4.00.)

The Standard Medical Directory of North America. 1903-4. Including a Directory of Practising Physicians in the United States of America, Canada, Cuba, Mexico and Central America. Lists of practitioners of the Specialties and Alphabetical index of all Physicians in North America. Also Directories of Medical Officers of the United States Army and Navy, Medical Societies, Medical Colleges, Medical Laws and Boards, Medical Publications (Books and Periodicals), Hospitals and Sanitariums. Mineral Springs, Drugs and Medicines, Medical and Surgical Products, Manufacturers and Life Insurance Companies. Chicago: G. P. Engelhard & Co. (Price, $10.00.)

Functional Diagnosis of Kidney Disease. With especial reference to Renal Surgery. Clinical experimental investigations by Drs. Leopold Casper and Paul Friederich Richter, Berlin. Translated by Dr. Robert C. Bryan, Richmond, Va., and Henry L. Sanford, Cleveland, O. Duodecimo, pp. 233. Philadelphia: P. Blakiston's Son & Co. 1903. (Price, $1.50.)

Nose and Throat Work for the General Practitioner. By George L. Richards, M.D., Otologist and Laryngologist, Fall River Union Hospital, Fall River, Mass. New York: International Journal of Surgery Co. 1903. (Price, $2.00.)

Physicial Diagnosis of Diseases of the Chest. By Richard C. Cabot, M.D., Physician to out-patients, Massachusetts General Hospital. Small octavo, pp. 335. One hundred and forty-seven illustrations. Second revised edition. New York: William Wood & Co. 1903. (Price, $2.50.)

A Textbook of Operative Surgery. Covering the Surgical Anatomy and Operative Technic Involved in the Operations of General Surgery. Written for Students and Practitioners. By Warren Stone Bickham, Phar. M., M.D., Assistant Instructor in Operative Surgery, College of Physicians and Surgeons, New York. Handsome octavo of 984 pages, with 559 illustrations, entirely original. Philadelphia, New York, London: W. B. Saunders & Company. 1903. (Cloth, $6.00 net; sheep or half morocco, $7.00 net.)

A Dictionary of Medical Science. Containing a full explanation of the various subjects and terms of Anatomy, Physiology, Medical Chemistry, Pharmacy, Pharmacology, Therapeutics, Medicine, Hygiene, Dietetics, Bacteriology, Pathology, Surgery, Ophthalmology, Otology, Laryngology, Dermatology, Gynecology, Obstetrics, Pediatrics, Medical Jurisprudence,

Dentistry, Veterinary Science, etc. By Robley Dunglison, M.D., LL.D., late Professor of Institutes of Medicine and Medical Jurisprudence in the Jefferson Medical College of Philadelphia. New (twenty-third) edition, thoroughly revised, with the pronunciation, accentuation and derivation of the terms, by Thomas L. Stedman, A.M., M.D., Fellow of the New York Academy of Medicine. In one imperial octavo volume of 1224 pages, with about 600 illustrations, including 85 full-page plates, mostly in colors, with thumb-letter index. Philadelphia and New York: Lea Brothers & Co. 1903. (Cloth, $8.00 net; leather, $9.00 net; half morocco, $9.50 net.)

A Textbook of Pathology. By Alfred Stengel, M.D., Professor of Clinical Medicine in the University of Pennsylvania. Octavo volume of 933 pages, with 349 text-illustrations, many in colors, and 7 full-page colored plates. Fourth edition, revised. Philadelphia, New York, London: W. B. Saunders & Company. 1903. (Cloth, $5.00 net; sheep or half morocco, $6.00 net.)

American Textbook of Surgery. For Practitioners and Students. Edited by William W. Keen, M.D., LL.D., F. R. C. S. (Hon.), Professor of the Principles of Surgery and of Clinical Surgery, Jefferson Medical College, Philadelphia; and J. William White, M.D., Professor of Surgery, University of Pennsylvania, Philadelphia. Fourth edition, thoroughly revised and greatly enlarged. Handsome octavo of 1363 pages, with 551 text-illustrations and 39 full-page plates, many in colors. Philadelphia, New York, London: W. B. Saunders & Company. 1903. (Cloth, $7.00 net; sheep or half morocco, $8.00 net.)

Clinical Examination of the Urine and Urinary Diagnosis. A Clinical Guide for the use of Practitioners and Students of Medicine and Surgery. By J. Bergen Ogden, M.D., formerly Instructor in Chemistry, Harvard University Medical School, Boston. Second revised edition. Handsome octavo volume of 418 pages, illustrated, including 11 plates, 9 of them in colors. Philadelphia, New York, London: W. B. Saunders & Company. 1903. (Cloth, $3.00 net.)

The Practical Medicine Series of Year Books. Ten volumes. Issued under the general editorial charge of Gustavus P. Head, M.D., Professor of Laryngology and Rhinology in the Chicago Post-Graduate Medical School. Volume IX. Physiology, Pathology, Bacteriology, Anatomy, Dictionary. Edited by W. A. Evans, M.D., Adolph Gehrmann, M.D., William Healy, M.D. Duodemico, pp. 233. Chicago: The Year Book Publishers. 1902. (Price, $1.25; entire series, $7.50.)

Modern Surgery: General and Operative. By John Chalmers DaCosta, M.D., Professor of the Principles of Surgery and of Clinical Surgery in the Jefferson Medical College, Philadelphia. Handsome octavo volume of 1099 pages, with over 700 illustrations, some in colors. Fourth edition, greatly enlarged and entirely reset. Philadelphia, New York, London: W. B. Saunders & Company. 1903. (Cloth, $5.00 net; sheep or half morocco, $6.00 net.)

Nervous and Mental Diseases. By Archibald Church, M.D., Professor of Nervous and Mental Diseases and Head of Neurological Department, Northwestern University Medical School; and Frederick Peterson, M.D., President New York State Commissioner in Lunacy; Chief of Clinic, Department of Nervous Diseases, College of Physicians and Surgeons, New York. Fourth edition, thoroughly revised and enlarged. Handsome octavo volume of 922 pages, with 338 illustrations. Philadelphia, New York, London: W. B. Saunders & Company. 1903. (Cloth, $5.00 net; sheep and half morocco, $6.00 net.)

A Textbook of the Practice of Medicine. By James M. Anders, M.D., Ph. D., LL.D., Professor of the Practice of Medicine and of Clinical Medicine, Medico-Chirurgical College, Philadelphia. Sixth edition, thoroughly revised. Handsome octavo volume of 1300 pages, fully illustrated.

Philadelphia, New York, London: W. B. Saunders & Company. 1903. (Cloth, $5.50 net; sheep or half morocco, $6.50 net.)

A Textbook of Obstetrics. By Barton Cooke Hirst, M.D., Professor of Obstetrics in the University of Pennsylvania. Handsome octavo, 900 pages, with 746 illustrations, 39 of them in colors. Philadelphia, New York, London: W. B. Saunders & Company. 1903. (Cloth, $5.00 net; sheep or half morocco, $6.00 net.)

A Textbook of Obstetrics. By J. Clarence Webster, M.D., (Edin.), F. R. C. P. E., F. R. S. E., Professor of Obstetrics and Gynecology, Rush Medical College, in Affiliation with the University of Chicago; Obstetrician and Gynecologist to the Presbysterian Hospital, Chicago; Obstetrician to the Chicago Lying-in Hospital and Dispensary, Chicago; etc., etc. Handsome octavo volume of 767 pages, with 383 illustrations, 23 in colors. Philadelphia, New York, London: W. B. Saunders & Company. 1903. (Cloth, $5.00 net; sheep and half morocco, $6.00 net.)

